# Multifaceted roles of SCRM/ICE1 in stomatal development, cold stress and beyond

**DOI:** 10.3389/fpls.2026.1808286

**Published:** 2026-04-01

**Authors:** Yongqiang Chen, Rui Zhang

**Affiliations:** 1 College of Horticulture and Forestry, Tarim University, Alar, China; 2 National and Local Joint Engineering Laboratory of Efficient and High-Quality Cultivation and Deep Processing Technology of Characteristic Fruit Trees in Southern Xinjiang, Alar, China; 3 Characteristic Forest and Fruit Technology Innovation Center of Southern Xinjiang in Xinjiang Production and Construction Corps (XPCC), Alar, China

**Keywords:** cold tolerance, ICE1, leaf senescence, reproductive development, SCRM, seed dormancy, stomatal development

## Abstract

The basic helix-loop-helix transcription factor SCREAM (SCRM), also known as INDUCER OF CBF EXPRESSION 1 (ICE1), functions as a regulatory hub that integrates environmental and hormonal signals to mediate diverse aspects of plant development and stress adaptation. Initially characterized for its roles in cold tolerance and stomatal development, recent studies have expanded its functional landscape to include reproductive development, seed dormancy, leaf senescence, and immune modulation. This review synthesizes current knowledge of SCRM/ICE1’s multifaceted functions across the plant life cycle, summarizing its molecular regulatory networks in development and stress responses respectively, and highlighting its central role as a regulatory node. By consolidating these dispersed findings, we discuss the balance between developmental programming and stress adaptation mediated by SCRM/ICE1, identify key questions for future research, and explore its potential applications in crop genetic improvement.

## 1 Introduction

Basic helix-loop-helix (bHLH) transcription factors are evolutionarily conserved proteins that regulate cell development in plants and animals (Dennis et al., 2019; Gao and Dubos, 2024). Their structure includes two functional regions: a DNA-binding basic region and a dimerization HLH region. *Arabidopsis* has 158 bHLH proteins, categorized into 26 subfamilies. Among these, INDUCER OF CBF EXPRESSION 1 (ICE1), a member of the IIIb bHLH subfamily, was first identified through a genetic screen for regulators of freezing tolerance (Chinnusamy et al., 2003; Pires and Dolan, 2010). ICE1 was shown to bind to the promoters of *CBF* genes and activate their expression under cold stress, establishing it as a master transcriptional regulator of cold acclimation (Chinnusamy et al., 2003; Kidokoro et al., 2022; Ding et al., 2024; Kim et al., 2024). Subsequent research revealed that ICE1 is allelic to SCREAM (SCRM), a key factor in stomatal development. SCRM/ICE1 acts as a direct heterodimerization partner for the stomatal lineage bHLH factors SPEECHLESS (SPCH), MUTE, and FAMA, which together orchestrate the stepwise progression of stomatal cell fate (Hofmann, 2008; Kanaoka et al., 2008; Pillitteri and Dong, 2013).

Beyond its roles in cold response and stomatal development, SCRM/ICE1 also plays critical roles in reproductive development, including pollen germination, anther dehiscence, embryo development and endosperm breakdown (Denay et al., 2014; Wei et al., 2018; Luo et al., 2024; Chen et al., 2025a). It integrates jasmonate (JA) signaling to regulate stamen development and male fertility (Li et al., 2025), and interacts with hormonal pathways to control seed dormancy and germination (Hu et al., 2019; MacGregor et al., 2019). Furthermore, SCRM/ICE1 acts as a negative regulator in leaf senescence, suppressing the process induced by dark and brassinosteroid (BR) treatments (Kim et al., 2020). Recent studies have also revealed its role in mediating crosstalk between cold stress and immune responses via salicylic acid (SA) signaling (Li et al., 2024). During the plant life cycle, SCRM/ICE1 participates in regulating different developmental stages through interactions with multiple hormones. These regulatory mechanisms enable SCRM/ICE1 to integrate environmental and endogenous signals across multiple biological contexts.

In this review, we systematically summarize the molecular mechanisms by which SCRM/ICE1 regulates diverse processes—including stomatal development, cold tolerance, reproduction, seed germination, and senescence—through its integration of environmental and hormonal signals. The functional versatility and regulatory complexity of ICE1 underscore its importance as a central node in plant development and stress adaptation, making it a compelling subject for both basic research and crop improvement.

## 2 SCRM/ICE1 in stomatal development

**Figure 1:**
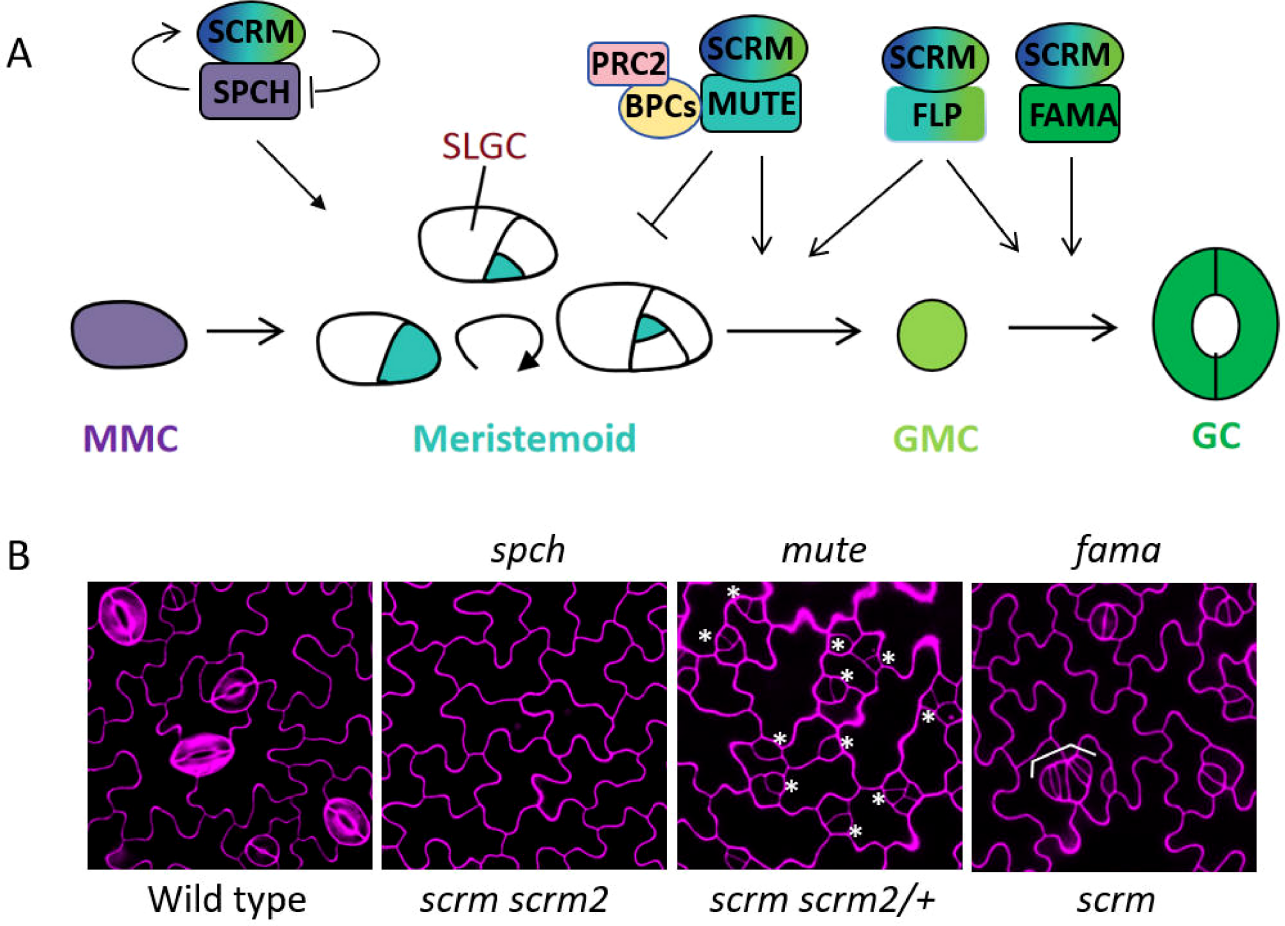
Role of Arabidopsis SCRM/ICE1 in stomatal development. **(A)** During the stomatal lineage, SCRM/ICE1 is activated by SPCH in meristemoid mother cells. Subsequently, SCRM/ICE1 and SPCH form heterodimers to promote asymmetric divisions, with SPCH maintaining high meristemoid activity and promoting its self-renewal. The accumulated SCRM/ICE1 then inhibits the expression of SPCH, forming a regulatory loop (SPCH-SCRM-SPCH) that ensures controlled proliferative divisions within 1~3 rounds. Upon MUTE expression, SCRM/ICE1 forms a complex with it to further facilitate the termination of proliferative division and meristemoid differentiation. In the proliferation stage, the MUTE-SCRM/ICE1 module, mediated by BPCs, terminates asymmetric division. This recruits the PRC2 complex to *SPCH* loci, leading to chromatin structure changes through repressive histone mark deposition (Me, H3K27me3). In the diagram, purple cells represent stomatal lineage precursors, blue cells denote late meristemoids and guard mother cells, and green cells signify immature and mature guard cells. **(B)** Double mutation of *SCRM/ICE1* and *SCRM2* recapitulates the phenotype of *spch*, an epidermis devoid of any stomatal lineages. Haploid mutation of *SCRM2* in *scrm* mutant exhibits a phenotype identical to *mute*, meristemoids undergoing excessive asymmetric divisions. Single mutation of *SCRM/ICE1* results in a weak *fama* phenotype due to the formation of extra parallel-aligned guard mother cells. Asterisks indicate arrested meristemoids, and brackets indicate guard mother cell-like tumors (Epidermal cells from plants of different ecotypes were stained with propidium iodide (magenta) and imaged using confocal microscopy).

Stomata are specialized structures on the plant epidermis, each consisting of two guard cells that form a central pore. They regulate gas exchange between the plant and environment. Stomatal development begins with an asymmetric division of a stem-cell-like precursor cell, followed by a single symmetric division that produces a pair of guard cells (Bergmann and Sack, 2007; Lau and Bergmann, 2012; Pillitteri and Torii, 2012; Pillitteri and Dong, 2013; Lee and Bergmann, 2019; Herrmann and Torii, 2021; Kim and Torii, 2023) (**Figure 1A**). Studies have shown that each cell-state transition and cell division in stomatal lineage are mediated by cell-type-specific transcription factors. Three bHLH proteins, SPCH, MUTE, and FAMA, drive the sequential steps of stomatal development including stomatal entry, commitment, and differentiation, respectively (Ohashi-Ito and Bergmann, 2006; MacAlister et al., 2007; Pillitteri et al., 2007). Two additional bHLH proteins, SCRM/ICE1 and SCRM2 can interact with SPCH, MUTE, and FAMA to specify the sequential actions during stomatal development (Kanaoka et al., 2008) (**Figure 1A**). Double mutation of *SCRM* and *SCRM2* recapitulates the phenotype of *spch*, an epidermis devoid of any stomatal lineages. Haploid mutation of *SCRM2* in *scrm* mutant exhibits a phenotype identical to *mute*, meristemoids undergoing excessive asymmetric divisions. Single mutation of *SCRM*, but not *SCRM2*, results in a weak *fama* phenotype due to the formation of extra parallel-aligned guard mother cells (Hofmann, 2008; Kanaoka et al., 2008; Larue and Zhang, 2019) (**Figure 1B**). Conversely, the gain-of-function mutant of *SCRM*, *scrm*-*D*, exhibits an epidermis solely made of stomata (Kanaoka et al., 2008). Why does this mutant exhibit such a severe stomatal developmental phenotype? In *scrm-D*, KRAAM is mutated to KHAAM, abolishing the interaction of SCRM with MITOGENACTIVATED PROTEIN KINASE 3/6 (MPK3/6), therefore the scrm-D protein cannot recruit MPK3/6 to interact with and modulate the stability of itself and SPCH. This leads to the accumulation of highly stabilized SPCH protein in *scrm-D*, thereby dramatically promoting stomatal development (Larue and Zhang, 2019; Putarjunan et al., 2019).

In *Arabidopsis* stomatal lineage, meristemoid undergoes limited rounds (typically 1~3 rounds) of asymmetric divisions before further differentiation, suggesting that the activities of proliferative asymmetric divisions are restricted and need to be terminated timely (Abrash and Bergmann, 2009). MUTE is first discovered to inhibit proliferative asymmetric divisions (Pillitteri et al., 2007). Recent studies have shown that SCRM is also involved in the termination of asymmetric cell divisions by directly repressing *SPCH* transcription in a MUTE-independent way (Chen et al., 2025b; Trosch, 2025). The single mutation of *scrm* leads to a delayed proliferation-to-differentiation transition phenotype with excessive asymmetric divisions, while overexpression of *SCRM* promotes the timely termination of proliferative divisions. Additionally, the expression levels of *SPCH* and its targeted genes are upregulated in *scrm* mutant and downregulated in *SCRM* overexpression plants (Chen et al., 2025b). Take together, since *SCRM* is activated by SPCH (Lau et al., 2014; Simmons and Bergmann, 2016), accumulated SCRM in turn inhibits the expression of *SPCH*, thus forming a regulatory loop of SPCH-SCRM-SPCH to maintain the balance between asymmetric divisions initiation and termination (Chen et al., 2025b) (**Figure 1A**).

The interaction between SCRM and MUTE is essential for the further differentiation of meristemoids. The C-terminal ACT like (ACTL) domain of SCRM is essential for MUTE-governed transition from meristemoid proliferation to differentiation (Seo et al., 2022). Mutations in the SCRM-ACTL domain specifically impair heterodimerization with MUTE and block the expression of MUTE’s direct target genes (Seo et al., 2022). The SCRM-MUTE complex is also recruited to chromatin by BASIC PENTACYSTEINE 1/2 (BPC1/2) transcription factors (**Figure 1A**). This recruitment occurs through binding to BBR/BPC (GAGA-repeat) motifs in a cell state-specific manner (Kooiker et al., 2005; Mu et al., 2017; Liu et al., 2020). BPC proteins recruit the POLYCOMB REPRESSIVE COMPLEX 2 (PRC2) complex to *SPCH* locus, which modifies chromatin structure by depositing the repressive histone mark H3K27me3, thus suppressing *SPCH* expression and terminating asymmetric cell divisions (Kim et al., 2022; Kim and Torii, 2023).

Recently, it has been reported that the MYB transcription factor FOUR LIPS (FLP), in addition to restricting guard mother cell division (Lai et al., 2005; Xie et al., 2010), is also involved in the transition from meristemoid to guard mother cell (Li et al., 2023). The *scrm flp* double mutants produced arrested meristemoids similar to *mute*, and SCRM physically interact with FLP (Li et al., 2023) (**Figure 1A**). Although it is not mentioned whether FLP is involved in the regulation of asymmetric cell divisions, SCRM may cooperate with FLP to terminate asymmetric divisions (MacAlister et al., 2007; Pillitteri et al., 2007; Han et al., 2022; Chen et al., 2025b). Furthermore, *FLP*, which is a direct target of MUTE (Han et al., 2018), may directly suppress asymmetric divisions in a manner similar to MUTE (Han et al., 2022).

The progress of stomatal development is highly susceptible to environmental influences (Pillitteri and Dong, 2013; Qi and Torii, 2018). SCRM acts as a key integrator of light signals into the stomatal developmental program. Under light conditions, the E3 ubiquitin ligase CONSTITUTIVE PHOTOMORPHOGENIC1 (COP1) activity is suppressed (Han et al., 2020; Nan and Hou, 2025), reducing COP1 mediated degradation of SCRM and leading to initiation of stomatal development. Thus, SCRM integrates light signaling into the core regulatory network of stomatal development, significantly influencing stomatal density (Lee et al., 2017; Qi and Torii, 2018). Does SCRM serve as a primary hub integrating other environmental cues into stomatal development? Addressing this question is essential for future research.

The functions of SCRMs have been extensively studied in *Arabidopsis*, and while their roles are largely conserved across species, certain functional divergences have also been observed. In rice, SCRMs have been reported to promote stomatal development through physical interactions with SPCH, MUTE, and FAMA (Wu et al., 2019). In *Brachypodium distachyon*, BdSCRM/BdICE1 and BdSCRM2 exhibit distinct functions: BdSCRM/BdICE1 is essential for the initiation of stomatal cell fate, whereas BdSCRM2 is required for the differentiation of stomatal complexes (Raissig et al., 2016). This functional divergence may be because BdSCRM/BdICE1 and BdSCRM2 are true paralogs (possibly from an ancient duplication) that fulfill specialized roles at different stages of stomatal development. In maize, *ZmICEb* is expressed in stomatal lineage cells and plays a functionally conserved role in regulating this process. Mutations in *ZmICEb* significantly enhanced drought tolerance and water use efficiency (WUE), while also mitigating yield loss under drought stress (Zhou et al., 2025). Stomata are present in nearly all major lineages of land plants, with liverworts being the only exception. In the moss *Physcomitrium patens*, MpSCRM1/2 regulate epidermal development and gametangiophore formation. The liverwort *SCRM/ICE1* genes partially restore stomatal developmental defects of the *atscrm1* mutant, suggesting that stomata across land plants share a common evolutionary origin (Chang et al., 2023).

## 3 SCRM/ICE1 in cold tolerance

Cold stress is a major factor influencing the geographical distribution and growth of plants. Low temperatures can cause significant damage to plants, leading to reduced crop productivity and quality. During cold acclimation, the physiological and metabolic status of plants undergoes substantial changes, accompanied by alterations in the expression of a large number of genes (Chinnusamy et al., 2007; Miura et al., 2011). The three C‐REPEAT/DRE BINDING FACTORs (CBF1-3), which belong to APETALA2/ETHYLENE-RESPONSIVE FACTOR (AP2/ERF) transcription factors, play an important role in cold acclimation by regulating the expression of a group of COLD-RESPONSIVE (COR) genes (Jaglo-Ottosen et al., 1998; Liu et al., 1998; Medina et al., 1999). SCRM/ICE1 was the first identified transcriptional activator of *CBF* genes. Loss of *SCRM/ICE1* function reduces plant tolerance to both chilling and freezing stresses, regardless of cold acclimation status. SCRM/ICE1 binds to the MYC recognition elements (CANNTG) in the promoters of *CBF1–3* and activates their expression under cold treatment (Chinnusamy et al., 2003; Liu et al., 2018; Tang et al., 2020; Chen et al., 2021).

**Figure 2:**
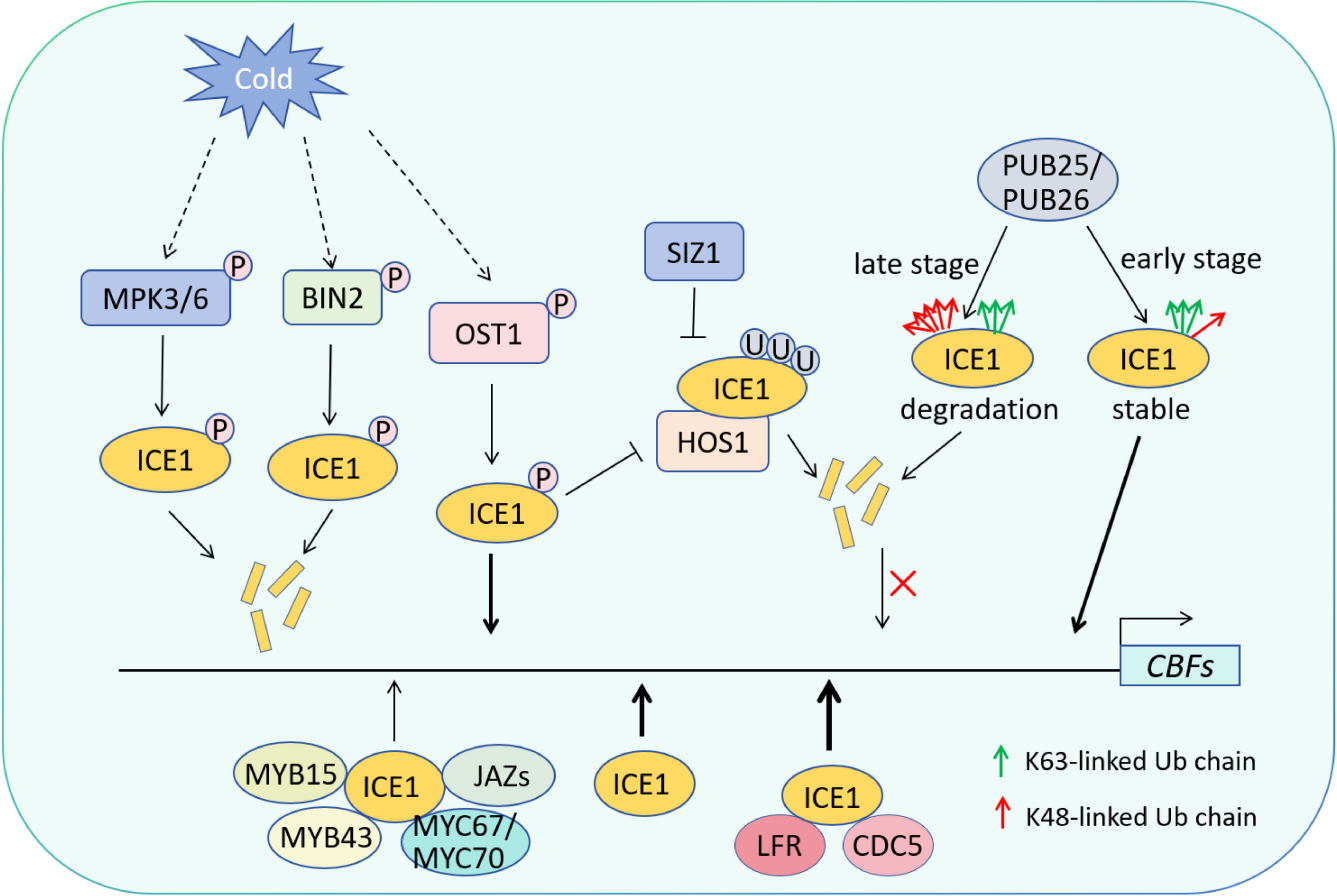
SCRM/ICE1 orchestrates a complex regulatory network to modulate cold tolerance. As a key transcriptional activator, SCRM/ICE1 binds to the promoters of *CBF* genes and activates their expression, thereby positively regulating the cold response. This regulatory process is finely tuned by multiple protein interactions: MYB43, MYB15, and JAZ proteins physically interact with SCRM/ICE1 and repress its transactivation activity on *CBF* promoters, thereby attenuating freezing tolerance. In contrast, the chromatin remodeler LFR associates with SCRM/ICE1 and enhances its binding to the *CBF3* promoter, further promoting cold-induced gene expression. Post-translational modifications play a critical role in dynamically controlling SCRM/ICE1 stability and activity. Upon cold exposure, activated MPK3/6 and BIN2 kinases phosphorylate SCRM/ICE1, promoting its ubiquitination and degradation. Conversely, the protein kinase OST1 is activated under cold stress and phosphorylates SCRM/ICE1, which disrupts its interaction with the E3 ubiquitin ligase HOS1, thereby stabilizing SCRM/ICE1. Additionally, the SUMO E3 ligase SIZ1 mediates sumoylation of SCRM/ICE1, enhancing its stability and transcriptional activity. The E3 ligases PUB25 and PUB26 further contribute to this multi-layered regulation by dynamically modulating SCRM/ICE1 stability through both K48- and K63-linked ubiquitination at different phases of the cold response.

As a crucial positive regulator of *CBFs*, SCRM/ICE1 has been extensively studied for its post-translational modifications (**Figure 2**). The RING finger protein HIGH EXPRESSION OF OSMOTICALLY RESPONSIVE GENE1 (HOS1), functioning as an E3 ubiquitin ligase, interacts with SCRM/ICE1 and promotes its degradation both *in vitro* and *in vivo*, thereby negatively regulating freezing tolerance (Dong et al., 2006). Unlike HOS1, SAP AND MIZ1 (SIZ1) acts as a SUMO E3 ligase that stabilizes SCRM/ICE1, enhancing freezing tolerance. Sumoylation of recombinant SCRM/ICE1 reduces polyubiquitination of the protein *in vitro*. SIZ1-dependent sumoylation of SCRM/ICE1 thus stabilizes and/or activates the protein, promoting *CBF3* expression and conferring cold tolerance (Miura et al., 2007). Furthermore, two E3 ligases, PLANT U-BOX 25 (PUB25) and PUB26, dynamically modulate the stability of the transcriptional regulator SCRM/ICE1 during different periods of cold stress by mediating both K48- and K63-linked ubiquitination. Shortly after cold exposure (1 h), K63-linked ubiquitination may stabilizes SCRM/ICE1, promoting *CBFs* expression. Later (6 h), increased K48-linked ubiquitination likely destabilizes SCRM/ICE1, attenuating the cold response (Wang et al., 2023).

OPEN STOMATA 1 (OST1) is a well-characterized Ser/Thr protein kinase central in ABA signaling (Mustilli et al., 2002). Under cold stress conditions, activated OST1 phosphorylates SCRM/ICE1 to enhance its stability, while simultaneously disrupting the HOS1-ICE1 interaction, thereby inhibiting HOS1-mediated SCRM/ICE1 degradation (Ding et al., 2015; Lang and Zhu, 2015; Zhan et al., 2015). Conversely, MPK3, MPK6, and BR INSENSITIVE2 (BIN2) interact with and phosphorylate ICE1 to promote its degradation, thereby negatively regulating freezing tolerance (Miura et al., 2011; Li et al., 2017a; Zhao et al., 2017; Ye et al., 2019). Consistent with this, the single mutant *mpk3*, *mpk6*, and double mutant *mpk3 mpk6* all exhibited freezing tolerance, while activation of MPK3/6 weakened freezing tolerance (Li et al., 2017a; Zhao et al., 2017; Liu and Zhou, 2018). Components of BR signaling positively regulate cold-induced *CBF* transcription. The transcription factors BRASSINAZOLE-RESISTANT 1 (BZR1), BRI1-EMS-SUPP RESSOR 1 (BES1), and CESTA (CES) are key positive regulatory factors in BR response, directly binding to *CBFs* promoters to promote their expression (Eremina et al., 2016; Li et al., 2017b). BIN2 is a key upstream kinase of these transcription factors. Under cold stress, BIN2 interacts with SCRM/ICE1 and undergoes phosphorylation, while promoting the interaction between SCRM/ICE1 and HOS1, thereby mediating the degradation of SCRM/ICE1 and negatively regulating freezing tolerance (Ye et al., 2019). These findings reveal a close interplay between cold stress and BR signaling.

In cold stress responses, the activity of SCRM/ICE1 is finely tuned by a sophisticated “phosphorylation-ubiquitination” regulatory switch. OST1-mediated phosphorylation stabilizes SCRM/ICE1 by disrupting its interaction with the E3 ubiquitin ligase HOS1 (Ding et al., 2015), thereby acting as a brake on SCRM/ICE1 degradation. In contrast, phosphorylation by MPK3/6 and BIN2 serves as an accelerator (Li et al., 2017a; Zhao et al., 2017; Ye et al., 2019), promoting SCRM/ICE1 turnover. The E3 ligases PUB25 and PUB26 further contribute to this regulatory complexity by mediating both stabilizing K63-linked and destabilizing K48-linked ubiquitination of SCRM/ICE1 at different stages of cold stress (Wang et al., 2023). This multi-layered control ensures a rapid yet transient cold response, preventing excessive energy expenditure. Interestingly, while MPK3/6-mediated phosphorylation of SCRM/ICE1 also modulates stomatal development by regulating the stability and activity of SPCH (Lampard et al., 2008; Putarjunan et al., 2019), the precise mechanisms controlling SCRM/ICE1 protein levels and activity during stomatal lineage progression remain largely unexplored.

Studies on SCRM/ICE1 interaction with other transcription factors and involvement in cold stress tolerance has gradually gained attention. Among them, transcription factors MYB15, MYC67/70, and MYB43 can all interact with SCRM/ICE1 and antagonize it, negatively regulating cold stress tolerance. MYB15, an R2R3-type transcription factor, directly binds to the promoters of *CBF* genes and represses their expression. The SCRM/ICE1 protein interacts with MYB15 and alleviates MYB15-mediated repression of *CBF* genes (Agarwal et al., 2006; Wang et al., 2023). Two MYC-type transcription factors, MYC67 and MYC70, interact with SCRM/ICE1 and participate in freezing stress regulation. The *myc67 myc70* double mutant exhibits enhanced freezing tolerance, whereas overexpression lines show a freezing-sensitive phenotype. As interacting partners of SCRM/ICE1, MYC67 and MYC70 may disrupt the binding of SCRM/ICE1 to cis-acting elements, thereby negatively regulating the expression of cold-responsive genes and freezing tolerance (Ohta et al., 2018). The transcription factor MYB43 directly binds to the promoters of *CBF* genes and represses their expression, functioning as a negative regulator of freezing tolerance. MYB43 physically interacts with SCRM/ICE1 and antagonizes its activity, forming a regulatory module that regulates the expression of *CBF* genes and plant’s freezing stress tolerance (Zheng et al., 2023). In contrast, LEAF AND FLOWER RELATED (LFR), a transcriptional regulatory component of the SWI/SNF chromatin remodeling complex, physically interacts with SCRM/ICE1 and directly promotes the transcriptional expression of *CBF3*. Chromatin immunoprecipitation assays reveal LFR’s association with SCRM/ICE1 chromatin, where it facilitates SCRM/ICE1 transcription. Importantly, the LFR-ICE1 interaction is functionally required for cold stress-induced activation of both *SCRM/ICE1* and *CBF3* expression (Ma et al., 2023). Furthermore, the R2R3 MYB transcription factor Cell Division Cycle 5 (CDC5) interacts with SCRM/ICE1 and regulates the expression of *CBF3* by recruiting RNA polymerase II. Overexpression of *SCRM/ICE1* can complements the freezing deficient phenotype of *cdc5* mutant, indicating that *SCRM/ICE1* acts epistatically to *CDC5* and both are positive regulators of cold tolerance (Xin et al., 2025).

Exogenous jasmonates significantly enhance plant freezing tolerance, while disruption of endogenous JA biosynthesis or signaling impairs cold resistance. Mechanistically, key JA signaling repressors, Jasmonate ZIM-domain (JAZ) proteins, including JAZ1 and JAZ4, directly interact with SCRM/ICE1. Specifically, JAZ1 and JAZ4 inhibit *SCRM/ICE1’s* transcriptional activity, leading to reduced expression of its downstream cold-responsive genes. Accordingly, overexpression of *JAZ1* or *JAZ4* attenuates freezing tolerance. Thus, JA acts as an essential upstream regulator of the ICEs-CBF pathway (Hu et al., 2013). Additionally, low-temperature stress enhances plant immunity, likely through the SA signaling pathway. Cold treatment promotes resistance to *Pseudomonas syringae* in an SCRM/ICE1-dependent manner. SCRM/ICE1 physically interacts with NONEXPRESSER OF PATHOGENESIS-RELATED GENES 1 (NPR1), a central regulator of SA signaling, which recruits SCRM/ICE1 to the *PATHOGENESIS-RELATED GENE 1* (*PR1*) promoter and enhances its transcriptional activity. Furthermore, cold stress strengthens the association among NPR1, TGACG-BINDING FACTOR 3 (TGA3), and SCRM/ICE1, boosting the transactivation capacity of the SCRM/ICE1-TGA3 complex on *PR1*. Thus, SCRM/ICE1 serves as a key integrator of cold-induced signaling and SA-mediated immunity, linking abiotic and biotic stress responses in plants (Li et al., 2024; Yang, 2024).

The function of SCRM/ICE1 in cold tolerance is conserved across plant species, primarily through activation of the CBF–COR pathway. For instance, in *Vitis amurensis* and *Solanum melongena*, SCRM/ICE1 enhances chilling tolerance by increasing antioxidant enzyme activities, proline content, and membrane stability (Dong et al., 2013; Zhou et al., 2020). In *Poncirus trifoliata* and *Pyrus ussuriensis*, it interacts with partners such as PuHHP1 or arginine decarboxylase to regulate polyamine synthesis and *DREB* expression (Huang et al., 2015a, 2015). Notably, some specific regulators fine-tune SCRM/ICE1 function. B-box (BBX) protein MdBBX37 in apple strengthens SCRM/ICE1-mediated activation of *CBF* genes (An et al., 2021), whereas in tomato, CALMODULIN6 (CaM6) inhibits SCRM/ICE1 activity and reduces cold tolerance (Lin et al., 2023). The Znf-CCCH protein PvC3H72 in switchgrass also operates upstream of the SCRM/ICE1–CBF module (Xie et al., 2019), illustrating diverse regulatory inputs into this conserved pathway.

## 4 Roles of SCRM/ICE1 during the *Arabidopsis* life cycle

Having detailed the molecular mechanisms of SCRM/ICE1 in stomatal development and cold stress response, we now broaden our perspective to its functions at the organismal level. The life cycle of *Arabidopsis*, like other plants, alternates between gametophytic and sporophytic phases. The gametophytic phase occurs within the flowers, involving the production of gametes and culminating in double fertilization, which gives rise to the embryo and endosperm. The sporophytic phase encompasses the majority of the plant’s life, beginning with seed germination and progressing through vegetative growth to reproductive development and senescence. As we will explore, the functions of SCRM/ICE1 are weaved into the progression of these critical phases, underscoring its role as a crucial regulator throughout the plant’s life cycle.

### 4.1 SCRM/ICE1 in pollen, embryo, and endosperm development

Plant sexual reproduction is a complex and highly organized process, which primarily includes floral organ formation, gametophyte development, pollination, fertilization, as well as seed development. Studies demonstrate that SCRM/ICE1 regulates multiple aspects of sexual reproduction, including stamen, embryo, and endosperm development. Compared to the wild-type, *ice1* mutants exhibit severe defects in sexual reproduction, manifesting as shorter filaments, impaired anther dehiscence, reduced pollen viability, and aberrant zygote development. These abnormalities result in failed fertilization, the formation of shortened siliques, and significantly elevated seed abortion rates (Wei et al., 2018; Luo et al., 2024; Li et al., 2025).

SCRM/ICE1 regulates male fertility through its control of water-related processes during anther development. A study reveals that defects in anther dehiscence and pollen germination are associated with impaired stomatal differentiation and altered expression of genes involved in water transport and ion exchange, disrupting hydration dynamics in the anther wall. This work highlights SCRM/ICE1 as a key regulator in male fertility through its influence on stomatal development and water movement within the anther (Wei et al., 2018). Furthermore, SCRM/ICE1 serves as an essential transcriptional activator of *QUA-QUINE STARCH* (*QQS*), enhancing pollen germination and viability via its interaction with INDETERMINATE DOMAIN 14 (IDD14). QQS shows increasing transcript levels during pollen development, peaking at maturity (Li et al., 2009). It regulates pollen surface lipid biosynthesis and promotes hydration and fertility. Notably, IDD14-mediated activation of *QQS* depends largely on SCRM/ICE1, and the assembly of the SCRM/ICE1-IDD14 complex is necessary for promoting pollen germination (Luo et al., 2024).

JA is essential for stamen development and sexual reproduction. Mutants of the JA receptor *COI1* exhibit typical stamen defects, such as shortened filaments, delayed anther dehiscence, and impaired pollen viability (Huang et al., 2014; Thireault et al., 2015; Huang et al., 2023). Notably, *SCRM/ICE1* knockout mutants phenocopy JA signaling deficiencies, underscoring its critical role in JA-mediated floral organ development and pollen formation. Mechanistically, JAZ proteins (JAZ1 and JAZ9), key JA signaling repressors, directly interact with SCRM/ICE1 to suppress the transcriptional activation of JA-responsive genes such as *MYB21*, *MYB24*, and *MYB108* (Li et al., 2025). These findings significantly advance our understanding of how SCRM/ICE1 integrates JA signaling to regulate reproductive development.

SCRM/ICE1 plays a pivotal role in embryonic development. SCRM/ICE1 serves as a downstream target of the embryonic ERECTA-YODA pathway and is essential for zygote polarization and the establishment of distinct cell identities during early embryogenesis (Lukowitz et al., 2004). Compared to wild-type, *scrm* mutants display shortened zygotes and impaired asymmetric division. Notably, unlike its role in stomatal development, SCRM/ICE1 activity in embryos is positively regulated by MPK-mediated phosphorylation. The ERECTA-YODA pathway enhances SCRM/ICE1 function through phosphorylation during early embryogenesis, contrasting sharply with the phosphorylation-dependent degradation mechanism observed in stomatal development (Putarjunan et al., 2019). Furthermore, genetic analyses reveal that SCRM/ICE1 acts in parallel with the transcription factor WRKY2 to activate *WUS-RELATED HOMEOBOX 8* (*WOX8*) expression in the zygote (Ueda et al., 2011, 2017). The identification of SCRM/ICE1 as a novel effector of the ERECTA-YODA pathway in embryogenesis underscores its versatile roles across diverse developmental and stress response pathways (Chen et al., 2025a).

During seed development, endosperm breakdown is coordinated with embryonic growth. In *Arabidopsis*, bHLH transcription factor ZHOUPI (ZOU) is specifically expressed in the endosperm cells. The *zou* mutant exhibits delayed embryo development and shortened suspensors. Its endosperm displays abnormal persistence throughout seed development, severely impairing embryonic growth (Yang et al., 2008). SCRM/ICE1 interacts with ZOU, via their bHLH domains. These two proteins co-express in the endosperm and function as heterodimers to regulate endosperm breakdown (Denay et al., 2014). In maize, the homologous gene *ZmICE1a* is also expressed in the entire endosperm. Loss of function of *ZmICE1a* reduces starch content and kernel weight. ZmICE1a is a key regulator of endosperm defense response and a coordinator of the defense-storage trade-off in endosperm development (Wang et al., 2024).

### 4.2 SCRM/ICE1 in seed dormancy and germination

Seed dormancy is a critical adaptive trait that promotes the formation of soil seed banks and prevents pre-harvest sprouting. In *Arabidopsis*, the endosperm-specific ZOU and SCRM/ICE1 play essential roles in regulating primary dormancy depth. Both *ice1* and *zou* single mutants exhibit enhanced seed dormancy, with the double mutant showing an additive effect, indicating their synergistic function. Mechanistically, SCRM/ICE1 directly binds to and represses *ABA INSENSITIVE 3* (*ABI3*) expression. Accordingly, *ABI3* expression is significantly upregulated in *ice1* mutants. This elevated *ABI3* level leads to increased ABA accumulation (MacGregor et al., 2019), which ultimately prolongs seed dormancy.

During seed germination, ABI5 serves as a key regulator of the ABA signaling pathway to suppress this process. Research has revealed that SCRM/ICE1 negatively regulates ABA responses by directly repressing the expression of ABI5’s target genes, *LATE EMBRYOGENESIS ABUNDANT6* (*EM6*) and *EM1* (Carles et al., 2002; Reeves et al., 2011). SCRM/ICE1 physically interacts with ABI5 and antagonizes its transcriptional activity. Furthermore, SCRM/ICE1 interacts with DELLA proteins, critical repressors of gibberellin (GA) signaling (Daviere and Achard, 2016). Genetic evidence demonstrates that SCRM/ICE1 disruption partially rescues the ABA hyposensitive phenotype of *della* mutants, indicating an antagonistic relationship between SCRM/ICE1 and DELLA proteins in ABA signaling. Consistently, DELLA proteins suppress both SCRM/ICE1’s transcriptional activity and its antagonistic effect on *ABI5*. Collectively, SCRM/ICE1 coordinates with ABI5 and DELLA proteins to fine-tune ABA signaling during seed germination (Hu et al., 2019).

### 4.3 SCRM/ICE1 in leaf senescence

Leaf senescence represents the final stage of leaf development in plants, which is a highly regulated physiological process. It involves the efficient mobilization and redirection of nutrients and energy from aging leaves. These resources are allocated to support the growth of new and developing tissues, such as young leaves and seeds, or are transferred to storage organs for future use (Guo et al., 2021). SCRM/ICE1 negatively regulates dark- and BR-induced leaf senescence through its interaction with ATBS1-INTERACTING FACTOR 2 (AIF2). AIF2, a non-DNA-binding bHLH transcription factor, functions to delay both dark- and BR-induced leaf senescence. Darkness triggers BR biosynthesis and subsequent BR signaling activation, leading to the positive regulator BZR1 of BR signaling to bind to the *AIF2* promoter (Kim et al., 2017). This binding represses *AIF2* transcription and accelerates senescence. Mechanistically, AIF2 interacts with SCRM/ICE1 to up-regulate *CBFs* expression while antagonistically down-regulating the early senescence-responsive gene *PHYTOCHROME INTERACTING FACTOR 4* (*PIF4*) (Song et al., 2014). This regulatory mechanism suppresses dark- and BR-triggered leaf senescence, thereby maintaining plant growth and enabling them to stay green longer (Kim et al., 2020).

## 5 Conclusion and perspective

Over the past two decades, research on SCRM/ICE1 has revealed its role not only as a key regulator of cold tolerance but also in stomatal development, reproductive biology, seed germination, and other critical biological processes. As a representative bHLH transcription factor, SCRM/ICE1 demonstrates remarkable functional diversity and regulatory complexity. It responds not only to cold stress but also participates in transducing various exogenous and endogenous signals including light, JA, ABA, and BR, establishing itself as a central regulatory node in plant development and environmental adaptation.

Stomatal development and drought tolerance are closely linked. Under prolonged drought stress, plants typically reduce stomatal density to minimize transpirational water loss, thereby enhancing drought resistance (Franks et al., 2015; Li and Le, 2026). For instance, mutations in *ZmICEb* significantly enhanced drought tolerance and water use WUE in maize, while also mitigating yield loss under drought stress (Zhou et al., 2025). This suggests that under water-limited conditions, reduced stomatal density resulting from *SCRM/ICE1* dysfunction may benefit plant survival and productivity. However, under normal conditions, the same reduction in stomatal density could lead to decreased carbon assimilation and ultimately lower crop yield, highlighting a trade-off between stress adaptation and growth. While the link between stomatal density and drought tolerance is mechanistically straightforward, the relationship between stomatal density and cold tolerance is far less clear. Cold stress primarily affects membrane fluidity, photosynthesis, and metabolic homeostasis (Kidokoro et al., 2022; Ding et al., 2024), with no obvious direct connection to stomatal density. Interestingly, both the gain-of-function mutant *scrm-D* (which exhibits high stomatal density) and loss-of-function *scrm* mutants (which exhibit low stomatal density) display enhanced sensitivity to cold stress. This observation suggests that freezing tolerance is not simply correlated with stomatal development, and that SCRM/ICE1 likely confers cold tolerance through mechanisms independent of its role in stomatal development.

Plant development is dynamically regulated by environmental cues and hormonal signals. SCRM/ICE1 is known to integrate light signaling into the core stomatal development pathway (Lee et al., 2017; Qi and Torii, 2018). This raises an important question: do other environmental factors, such as drought, also modulate stomatal development through SCRM/ICE1? Long-term drought stress reduces stomatal density, a process partly mediated by ABA (Yang et al., 2022). Notably, SCRM/ICE1 interacts with ABI5 to fine-tune ABA signaling during seed germination (Hu et al., 2019), suggesting a potential link between SCRM/ICE1 and ABA in drought responses. Whether ABA directly engages SCRM/ICE1 to regulate drought-induced stomatal development changes remains an open question worthy of investigation. Although cross-regulatory networks between SCRM/ICE1 and multiple hormone signaling pathways have been reported, the underlying molecular mechanisms remain poorly understood. For instance, how SCRM/ICE1 selectively interacts with different hormone signaling components in a tissue- or stage-specific manner, requires systematic investigation. Future studies employing approaches such as single-cell RNA sequencing (scRNA-seq), and single-cell Assay for Transposase-Accessible Chromatin using sequencing (scATAC-seq) will help elucidate the context-dependent interaction networks of SCRM/ICE1, thereby revealing the molecular basis of its functional versatility across diverse developmental and stress-responsive processes.

The role of SCRM/ICE1 in cold tolerance is well-established, but its functions under other types of stress await systematic exploration. Emerging evidence suggests that SCRM/ICE1 is also involved in responses to drought and salt stress. For instance, overexpression of *CdICE1* from chrysanthemum or *SlICE1a* from tomato significantly enhances drought and salt tolerance in transgenic plants (Chen et al., 2012; Feng et al., 2013), likely through activation of the CBF/DREB pathway and promotion of osmoprotectant accumulation. Furthermore, SCRM/ICE1 regulates the expression of *BON1-ASSOCIATED PROTEIN1* (*BAP1)* under moderately low temperatures (Zhu et al., 2011), indirectly influencing plant immune responses, suggesting its role in temperature-immune cross-talk. According to reports, ChIP-seq data indicates that SCRM/ICE1 is enriched at the promoters of stomatal movement related genes and wounding responsive genes (Tang et al., 2020). These findings imply that SCRM/ICE1 may have broader regulatory functions in multiple stress responses, highlighting its potential as a target for stress-resilience breeding.
